# Challenges and opportunities for comparative studies of survival rates: An example with male pinnipeds

**DOI:** 10.1002/ece3.7627

**Published:** 2021-05-08

**Authors:** Jamie L. Brusa, Jay J. Rotella, Katharine M. Banner, Patrick R. Hutchins

**Affiliations:** ^1^ Department of Ecology Montana State University Bozeman MT USA; ^2^ School of Environmental and Forest Sciences University of Washington Seattle WA USA; ^3^ Department of Mathematical Sciences Montana State University Bozeman MT USA

**Keywords:** comparative analysis, life‐history evolution, phylogenetic uncertainty, pinnipeds, survival rates

## Abstract

Survival rates are a central component of life‐history strategies of large vertebrate species. However, comparative studies seldom investigate interspecific variation in survival rates with respect to other life‐history traits, especially for males. The lack of such studies could be due to the challenges associated with obtaining reliable datasets, incorporating information on the 0–1 probability scale, or dealing with several types of measurement error in life‐history traits, which can be a computationally intensive process that is often absent in comparative studies. We present a quantitative approach using a Bayesian phylogenetically controlled regression with the flexibility to incorporate uncertainty in estimated survival rates and quantitative life‐history traits while considering genetic similarity among species and uncertainty in relatedness. As with any comparative analysis, our approach makes several assumptions regarding the generalizability and comparability of empirical data from separate studies. Our model is versatile in that it can be applied to any species group of interest and include any life‐history traits as covariates. We used an unbiased simulation framework to provide “proof of concept” for our model and applied a slightly richer model to a real data example for pinnipeds. Pinnipeds are an excellent taxonomic group for comparative analysis, but survival rate data are scarce. Our work elucidates the challenges associated with addressing important questions related to broader ecological life‐history patterns and how survival–reproduction trade‐offs might shape evolutionary histories of extant taxa. Specifically, we underscore the importance of having high‐quality estimates of age‐specific survival rates and information on other life‐history traits that reasonably characterize a species for accurately comparing across species.

## Graphical abstract

We present a Bayesian phylogenetically controlled generalized least squares model for comparative analyses and apply our model to real data of male pinnipeds. Male pinnipeds exhibit large interspecies variation in adult survival rates.

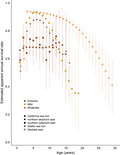

## 1 INTRODUCTION

The power of basic science lies in the comparison of similar empirical studies, and the comparative method can be useful for contextualizing broad hypotheses and identifying patterns across different studies (Adams, 2008; Freckleton, 2009; Stearns, 1983). Ecological and evolutionary meta‐analyses make several assumptions about data mined from empirical studies including independence of data from different studies, reasonable inclusion criteria for datasets given incomplete data reporting, comparability of data across studies, representativeness of data across a taxonomic group, and that available data for a specific species are representative of that species (Adams, 2008; Freckleton, 2009; Gurevitch & Hedges, 1999; Nakagawa et al., 2017; Noble et al., 2017; Pagel, 1999). A meta‐analysis is a statistical tool that specifically evaluates effect sizes calculated from parameters (or functions of parameters) associated with variables that represent the “size” of the relationship of interest (or “effect”) based on continuous data, binary data, or correlations (Vetter et al., 2013). Comparative analyses are similar to meta‐analyses but do not adhere to the requirement of comparing effect sizes (e.g., magnitude of change a covariate has with respect to the response variable, proportion of change in response variable from the treatment groups compared with the control groups) across studies (Felsenstein, 1985; Koricheva & Gurevitch, 2014; Vetter et al., 2013). As such, ecological and evolutionary comparative studies rely more heavily on the assumption that available data across a taxonomic group are representative of the taxonomic group. Additionally, they often assume that values gleaned from empirical studies are known without measurement error, but ignoring measurement error can lead to underestimating covariation between life‐history traits (Ives et al., 2007). Hierarchical models that incorporate variances and/or standard errors can better reflect this source of uncertainty in comparative data. Hierarchical models can incorporate phylogeny (specifying correlation structure among species when nonindependence issues are caused by species relatedness) to handle lack of independence in the data (Adams, 2008; Freckleton et al., 2002; Nakagawa et al., 2017). As ancestral traits can be maintained through several recent nodes of a phylogeny from phylogenetic inertia, it is important to account for a lack of independence across related species (Pagel, 1999). Further, ecologists have recently started incorporating measurement error (Hansen & Bartoszek, 2012; Ives et al., 2007) and phylogenetic uncertainty regarding tree topology or branch lengths (de Villemereuil et al., 2012) in comparative studies.

Comparative methods that evaluate variation in male survival rates across species can serve as a useful method for addressing a variety of life‐history evolution questions. Males of some species have unique energy investments related to growth and precopulatory or postcopulatory competition that might affect survival. Survival rates for juveniles and adults are fundamental characteristics of fitness and influence the diversity of life‐history strategies observed across iteroparous vertebrate species (Berta et al., 2018; Eberhardt, 1985; Gaillard & Yoccoz, 2003; Langtimm et al., 1998; Lemaître et al., 2015; Promislow & Harvey, 1990; Toïgo & Gaillard, 2003). However, few studies have investigated the connection between various life‐history strategies and survival rates in monophyletic groups, especially in long‐lived vertebrates (Sacher, 1978), and the relationships among life‐history traits seem to be better understood in females than males (Festa‐Bianchet, 2012). Because it is challenging to obtain sex‐ and age‐specific survival data in long‐lived vertebrate species (Hupman et al., 2018; Kanive et al., 2019; Lebreton et al., 1992), empirical data on survival rates for multiple species in a monophyletic group are limited. For groups with adequate data, information is also needed on measurement errors in life‐history traits (either through pseudoreplication or through standard errors on measurements from a single study) and evolutionary pathways so that uncertainties in these measurements can be incorporated into the comparative analyses (Ives et al., 2007; Revell & Reynolds, 2012; de Villemereuil et al., 2012).

Here, we present a model to demonstrate how to incorporate uncertainty in a response variable on the probability scale while also accounting for phylogeny for comparative analysis. We anticipate our model to be especially useful for addressing life‐history evolution questions pertaining to variation in rates of survival or reproduction. We provide results for simulated data to provide a “proof of concept” for the model, demonstrating its ability to recover assumed relationships engineered through the data‐generating process. Finally, we use real data from the suborder and monophyletic group, Pinnipedia, to preliminarily explore possible life‐history trait trade‐offs related to this taxonomic group and discuss limitations of data deficiencies through the application of our model to these real data. To further this avenue of research, we provide an organized compilation of currently available life‐history trait data for males of pinniped species. We also present recommendations for future comparative studies aimed at investigating relationships between life‐history traits, specifically the importance of more coordinated data collection efforts.

Pinnipedia serves as an interesting taxonomic group to investigate various relationships between age‐specific survival and reproductive traits. Pinniped species have a cosmopolitan distribution, represent a wide range of polygynous mating strategies, and exhibit diverse sexual selection in the form of body size (Ferguson & Higdon, 2006; Krüger et al., 2014; Le Boeuf, 1991; Stirling, 1983). Male pinnipeds also tend to undergo physiological and/or behavioral changes years after reaching sexual maturity. Greater selection for longevity is expected to occur when traits are differentially expressed at sexual maturity and social maturity—defined here as the mean age at which males begin to successfully engage in reproductive activity and/or sire offspring (Bonduriansky et al., 2008). Finally, Fitzpatrick et al. (2012) recently found that pinniped species tend to invest either in body mass, a precopulatory trait, or testes mass and baculum length, which are postcopulatory traits. An interesting advancement of the findings of Fitzpatrick et al. (2012) would be to investigate relationships between precopulatory and postcopulatory traits and other life‐history traits, such as survival rates.

Below, we detail potential covariates for our modeling framework that could address key questions in life‐history trait evolution, such as how do survival rates at different ages or life stages relate to 1) investment in precopulatory traits, 2) investment in postcopulatory traits, 3) age at sexual and social maturity, and 4) body length. In the following subsections, we provide the biological motivation for the aforementioned life‐history traits as applied to pinnipeds. Although our focus here is on pinnipeds, our modeling framework and our proposed life‐history trait evolution questions are also appropriate for other well‐studied taxonomic groups (e.g., ungulates).

### 1.1 Body size

Body mass typically serves as a useful indicator for the slow–fast life‐history continuum because larger body sizes tend to correlate with longer lifespans and higher survival rates (Gaillard et al., 2003; Promislow & Harvey, 1990), and achieving larger bodies can prove useful when living off of energy reserves when fasting during the mating season (Bartholomew, 1970; Le Boeuf, 1991). However, acquiring and maintaining energy sufficient for large bodies can become challenging in times of limited resources (Toïgo & Gaillard, 2003). In mammalian species, body mass can fluctuate to a great extent throughout the year, among years, with age, and among individuals of a species (Gaillard et al., 2003). Additionally, blubber concentrations can vary with season in pinnipeds (McLaren, 1993), and body mass metrics for all pinniped species are not prevalent enough for standardization to account for the variation associated with season, year, age, and individual. For these reasons, comparing body mass among pinniped species is inappropriate, but comparisons of body length can be informative (McLaren, 1993). We chose to use standard body length as a proxy for body size.

### 1.2 Mating system

Mating system can be used as a proxy for precopulatory traits. The range in the degree of polygyny across pinniped species exceeds that for most other taxonomic groups of mammals or birds and extends from a single mate during the breeding season to harems reaching as many as 100 females per male (Bartholomew, 1970; Bleu et al., 2016; Liker & Székely, 2005; Promislow, 1992; Stirling, 1983). In pinnipeds, mating systems can be defined using the following definitions by Le Boeuf (1991): mild polygyny as males siring 2–5 offspring per breeding season, moderate polygyny as males siring 6–15 offspring per breeding season, and extreme polygyny with males siring 16 or more offspring per breeding season. A high degree of male–male competition tends to accompany extreme polygyny and often results in lower longevity relative to species with lesser degrees of polygyny or monogamy (Clutton‐Brock & Isvaran, 2007; Tidière et al., 2015). However, Gaillard et al. (2003) found that the ratio of male mortalities to female mortalities was higher for populations with weak polygyny than strong polygyny in ungulates. Their finding suggests that a high degree of polygyny is not always a prerequisite for a reproduction–survival trade‐off in ungulates, which might also be the case for pinnipeds.

### 1.3 Sperm competition

We evaluated baculum length as a proxy for sperm competition. Baculum length has been found to vary independently of body length (Ramm, 2007). A larger baculum likely evolved in response to females mating with multiple males in a single breeding season because a baculum can facilitate sperm competition (Miller et al., 2000). However, baculum size also correlates with the copulation environment (terrestrial with dry bodies or aquatic or ice with wet bodies, Scheffer & Kenyon, 1963), and logistical properties for successful copulation might have also helped drive selection for baculum size. Given that females choose the frequency with which they mate, males with sperm that can move through the female reproductive tract with greater efficiency would have an advantage (Parker et al., 2012). The size and shape of the baculum in pinnipeds are also likely important for stimulating the female reproductive tract (Miller et al., 1998), which can add further selection pressure for a larger baculum.

As allocation of energy to sperm competition decreases the available energy for both precopulatory trait investment (e.g., large body size, weaponry) and somatic maintenance, greater investment in sperm competition might come with reduced investment in precopulatory traits and/or a cost to survival (Parker et al., 2012). Investing in both precopulatory and postcopulatory traits can increase the reproductive competitiveness of males in species that do not monopolize access to females, but exclusive investment in precopulatory traits in lieu of postcopulatory investment seems common for species in which males can restrict their competitor's access to females (Lüpold et al., 2014). Pinnipeds illustrate this pattern in that species that form harems have smaller bacula than species that do not form harems (Fitzpatrick et al., 2012).

## 2 MATERIALS AND METHODS

### 2.1 Bayesian measurement error model framework

To provide a framework for investigating the relationships between stage‐specific survival rates and other life‐history traits in future comparative studies, we present a phylogenetically controlled linear regression model in a Bayesian framework (similar to that presented by de Villemereuil et al. 2012) that can be written in the JAGS language (Plummer, 2003). We fit our model using the packages rjags (Plummer et al., 2016) and runjags for R (Denwood & Plummer, 2016). We use a simple linear model with standard length as a covariate to illustrate our general modeling framework. We explored model performance using simulated data and applied our model to real pinniped data. Our model incorporates one source of uncertainty in average survival and average standard body length estimates (i.e., standard error). Our model also incorporates uncertainty in the topology and branch lengths of the phylogenetic tree that represents the phylogeny of the species in our study. Unless noted otherwise, we used R Statistical Software version 3.5 (R Core Team, 2018) for all of our analyses.

Incorporating measurement error in comparative phylogenetic analyses is rare even though it can yield better parameter estimates and better detect phylogenetic signal (Ives et al., 2007). We adjusted the methodology presented by de Villemereuil et al., (2012) to include measurement error in survival rates when standard errors (SEs) are only reported on the probability scale (i.e., SE on the logit scale is often not reported). Specifically, we used the beta distribution parameterized for regression (Ferrari & Cribari‐Neto, 2004) to assume the observed survival rate for a particular species was a beta random variable with mean equal to the latent (true) survival probability and standard deviation close to the estimated standard error for each population. That is, the observed data were used to help specify the one unknown parameter in the species‐specific prior distribution for the observed survival rate, where the mean is assumed to be the true survival probability. The beta distribution has been used in a similar fashion to account for measurement error in imperfectly observed plant cover data (See Irvine et al., 2019). We assumed classical additive Berkson measurement error (Berkson, 1950) for average standard body length and used a normal distribution for the general species‐wide standard body length estimate, as it is reasonable to assume average body lengths will be approximately normally distributed. Specifically, the estimated/observed average standard body length for a species was assumed to be a normal random variable with mean equal to true standard body length for that species and standard deviation equal to the standard error that came from the empirical study estimating standard body length for that species.

To specify the model, let *Y_i_* be the true logit‐transformed overall survival rate for species *i* (*i = *1,2,…, *n_i_*_,_ and *n_i_* is the number of species in the *j^th^* stage, *j* = yearling, sexual maturity, social maturity), *W_i_* be the *observed* average standard body length, and *X_i_* be the true average standard body length of species *i*. Then, the logit‐linear regression model can be written as, $Y_{n_{j\;}\times \;1\;}∼\;MVN_{n_{j}\;\times \;1}\left(\alpha +\;\beta X_{n_{j}\;\times \;1}\;,\;\sigma_{\epsilon }^{2}\Sigma_{k}\right)$ where Σ_k_ is the *n_j_* × *n_j_* scaled variance–covariance matrix (or correlation matrix) calculated from the k^th^ (*k* = 1,2,…,100) phylogenetic tree as described by de Villemereuil et al. (2012), and a uniform categorical prior was used to provide equal prior probability to each candidate correlation matrix Σ_k_ (associated with one of the 100 phylogenetic trees). Weakly informative normal (0, σ^2^ = 4) priors were used on the partial regression coefficients (*α* and *β*) to constrain the estimates within a reasonable range on the logit scale (i.e., between −10 and 10), and a weakly informative gamma(1, 1) prior was used for the residual precision, $\sigma_{\epsilon }^{‐2}$ to help reduce autocorrelation in the MCMC (as described by de Villemereuil et al., 2012). The Berkson measurement error on the average body lengths was incorporated assuming $W_{i}|X_{i}\;∼\;N\left(X_{i},\;\sigma_{\mathrm{ui}}^{2}\right)$ and that the true average species lengths can be assumed to have come from a normal population distribution (i.e., assumes exchangeability), $X\;∼\;MVN_{n_{j}}\left(\mu_{x}\;,\;\sigma_{x}\Sigma_{k}\right)$ with a uniform prior on the population mean between 100 and 700, $\mu_{x}\;∼\;\mathrm{Uniform}\left(100,\;700\right)$ correlation structure equal to that specified by the phylogenetic tree, Σ_k_ and an inverse gamma prior on the common variance $\sigma_{x}^{‐2}\;∼\;\mathrm{gamma}\left(1,\;1\right)$. We selected the prior for the standard length based on the mean standard lengths observed across species; the shortest species in our dataset was 144 cm, and the longest species was 540 cm. As such, we deem standard body lengths shorter than 100 cm or longer than 700 cm to be impossible. We chose a uniform prior because this distribution made biological sense and would restrict the Markov Chain Monte Carlo (MCMC) sampling to only reasonable values for our specific variable of standard length. For future use of our model, researchers should select the distribution that best fits their covariates with regard to the question they are investigating. For our study, the joint distribution for the average standard body length might be improved by setting each species to its own prior with an appropriate distribution to match standard body lengths specific to that species and allowing each species to have its own posterior distribution that would better reflect the variation present in each species rather than including the variation across species. Measuring the phylogenetic influence of standard body length can help elucidate the potential of the standard body lengths of pinnipeds coming from a common distribution by ruling out the possibility if there is no phylogenetic influence on standard body length. The measurement error variance for the *i^th^* species ($\sigma_{\mathrm{ui}}^{2}$) must be provided as data (or could be estimated from the data if more than one observation per species is taken into account), and it was assumed to be equal to the squared standard error estimate for species *i*.

Measurement error information is often not available for the logit survival rates, as was the case for our real pinniped data. For our model, it was induced on the probability scale by letting *p_i_* = logit^‐1^(*Y_i_*), and assuming *D_i_*|*p_i_* ~Beta(*p_i_ϕ_i_*, *p_i_*(1–*p_i_*) *ϕ_i_*). That is, we let *p_i_* be the true, unknown survival rate for species *i* and specify *ϕ_i_*, a function of the typical beta distribution parameters (*ϕ_i_*=*α_i_* + *β_i_*), where *α* and *β* were set to reflect a Beta(*α_i_*, *β_i_*) distribution with mean and standard deviation similar to the observed survival rates and standard errors for species *i* (see Appendix S1 Example code for details and model code).

We constructed phylogenetic trees for the species we included in our analyses and incorporated uncertainty in branch lengths (de Villemereuil et al., 2012) to account for any lack of independence in life‐history traits from shared ancestry (Adams, 2008; Chamberlain et al., 2012; Felsenstein, 1985). Nucleotide sequences were retrieved from the NCBI nucleotide database using the rentrez package for R version 3.6 (R core Team, 2019). Up to, but no more than, the first 50 records for each of the 33 mitochondrial genes for each pinniped species in the model were retrieved. Species representation within each gene varied between 0 and 50 individual records (Table S1). Sequences were aligned with ClustalW using the msa package for R (Bodenhofer et al., 2015), and the most likely nucleotide substitution model for each gene was determined with the *modelTest* function of the phangorn package for R (Schliep, 2011). Internal and terminal gaps were both represented with a dashed line. Phylogenetic species supertrees were generated using the *BEAST method (Heled & Drummond, 2010). Briefly, BEAUti version 2.6.1 was used to create input files for all genes and species, and nucleotide substitution models were assigned for each gene according to the *modelTest* output. An estimated random local clock was set as the evolutionary rate prior for all genes, and an estimated Yule model was used as the tree prior. Species for which there were no representative sequences for a gene were simply entered in the alignment series as “N” ambiguities to satisfy the requirements of the BEAST program. BEAST version 2.6.1 was used to create two MCMC chains sampled every 1 × 10^4^ generations for 1.74 × 10^8^ generations. Convergence and chain diagnostics were visualized and assessed with the rwty package for R (Warren et al., 2017). The average standard deviation of split frequencies ended at 0.003 (95% CI = 0.000, 0.020). The pseudo‐estimated sample size (pseudo‐ESS, Lanfear et al., 2016) of the tree topology from the last 1,000 trees sampled from each chain was 176 (95% CI = 138, 340). Using the *vcv* function of the ape package for R (Paradis & Schliep, 2019), variance–covariance matrices were generated from a random selection of 100 of the last 1,000 trees sampled from the posterior distribution of each of the two MCMC runs. Using at least 100 trees with associated uncertainty estimates can provide regression parameter estimates for life‐history trait models with greater precision and reduce the frequency of making a type I error (de Villemereuil et al., 2012).

For our example, we incorporated the phylogenetic information into our model by using a known variance–covariance structure for the survival probabilities and standard body length by assuming it is equal to the corresponding particular phylogenetic tree. To incorporate uncertainty in the tree, we allowed the sampler to explore multiple trees (each with equal prior probability of being the “true tree”; that is, we introduced a discrete uniform prior on Σ_k_ with probability 1/100). We adjusted for phylogenetic influence of standard body length by incorporating Pagel's λ; a λ value of 0 indicates phylogenetic independence, and a λ value of 1 indicates strong phylogenetic dependence assuming Brownian motion (Freckleton et al., 2002; Pagel, 1999). We specified an uninformative uniform prior on λ, uniform(0, 1), because the whole range of lambda values is plausible within any comparative study.

### 2.2 “Proof‐of‐concept” simulation study

We used an unbiased simulation framework to demonstrate the ability of our model to estimate relationships between covariates of interest and median survival probability with nominal coverage and negligible bias. For simplicity and computational feasibility, we assumed one consensus tree for the 34 extant pinniped species (i.e., we did not allow for uncertainty in the phylogenetic structure of the tree in our simulation). We wrote a data‐generating function to produce datasets with a single male survival rate at the age of social maturity and centered and scaled standard body lengths for the 34 extant pinniped species. Survival rates and centered standard body lengths were assumed to be observed with error. True standard lengths (X) were generated as multivariate normal random variables with mean of 0 and variance–covariance matrix calculated from shared phylogenies for each species multiplied by a common variance of 0.25 to reflect a scaled covariate with standard deviation 0.5 (mathematically, $X_{n\;\times \;1}\;∼\;MVN(0_{n\;\times \;1},\;\sigma_{x}^{2}\Sigma_{n\;\times \;n}$). Observed standard lengths (W|X) were generated conditional on the true values with normal measurement error variance of $\sigma_{\mathrm{ui}}^{2}$(mathematically, $W_{i}|X_{i}\;∼\;N(X_{i},\;\sigma_{\mathrm{ui}}^{2}$), *i* = 1, 2, …, n). Measurement error variances were the same for all simulated datasets, and they were set by calculating 10% of |*X_i_*| for each species, reflecting more error than what was observed in the real data. We assumed a logit‐linear relationship between true survival rate and standard body length such that the true logit‐transformed survival rates were generated conditional on the true standard body lengths $Y=logit\left(p_{34\;\times \;1}\right)=\;\alpha +\beta X_{34\;\times \;1}+\;\varepsilon_{34\;\times \;1};\;\varepsilon_{34\;\times \;1}\;∼\;MVN\left(0_{34\;\times \;1},\;\sigma_{\varepsilon }^{2}\Sigma_{34\;\times \;34}\right)$). We chose values for the intercept and slope terms to represent a realistic hypothetical relationship between adult survival rate for male pinnipeds and standard body length. Specifically, we set α=0.2, implying a survival probability of about 0.55 for a male with average standard length, and a fixed coefficient for standard length (β = −1) to represent a relationship such that as standard body length increases, survival rate decreases in male pinnipeds. We assumed a residual standard error of $\sigma_{\varepsilon }$ = 0.55. We generated observed survival rates condition on the true survival rates using beta measurement error ($D_{i}|p_{i}\;∼\;Beta(p_{i}\phi_{i}\left(1‐p_{i}\right)$), where the ϕparameter was set to induce a beta distribution centered at *p_i_* with variance close to the squared standard errors for survival rates. Standard errors for survival rates were assumed to be known and generated from a uniform(0.01, 0.03), which reflected similar standard errors to those observed in the real data. A single random tree topology with 34 tips was generated with the *rtree* function of the ape package for R (Paradis & Schliep, 2019). We wrote a simulation wrapper to generate a dataset according to the process described above and then fit a Bayesian model described in the previous section to the simulated data with two slight modifications. As described above, we did not allow for uncertainty in the phylogenetic relatedness of the 34 species, and because the latent Xs were centered and scaled, we assumed a known prior mean of 0 and variance of 0.25.

Tuning revealed adequate convergence of the MCMC when 3 chains were run with the settings adapt = 5,000, burn‐in = 15,000, thin = 4, and sample = 35,000. We ran 100 iterations of this simulation, and for each parameter, we computed the posterior mean, 95% credible interval, number of effective samples, and Gelman–Rubin $\hat{R}$diagnostic. We inspected MCMC convergence using the coda package for R (Plummer et al., 2006) and ggmcmc package for R (Fernández‐i‐Marín, 2016) to observe Geweke convergence diagnostics (Geweke, 1991), trace plots, and calculate $\hat{R}$(Gelman & Rubin, 1992) for each monitored parameter. To assess the goodness of fit for our model, we created residual plots and plots to compare the observed data values and values predicted from the models (Conn et al., 2018). Additional goodness‐of‐fit testing can be performed following the guidelines by de Villemereuil et al. (2012). We evaluated the 95% credible interval coverage across the 100 iterations (see Appendix for additional details).

Future use of our modeling framework could include multiple observations per species and additional covariates (e.g., mating system or baculum length). To incorporate repeated measures of survival rates for the same species and estimate measurement error from the data, a hierarchical structure at the species level can be added to the model as in Lemaître et al. (2020). However, this approach assumes that all survival estimates for a particular species are exchangeable (reasonably modeled as having come from one common distribution), which will require assessment. Additionally, as different studies might have used different methods to estimate survival rates, it is important to consider the potential consequences of including multiple observations per species for making inferences.

### 2.3 Pinniped data collection

Applying consistent selection criteria for comparative studies limits the pool of data for comparative studies and, thus, the scope of inference. To emphasize the importance of defining inclusion criteria aimed to include life‐history trait estimates that represent each pinniped species as a whole, we outline our selection criteria in the next section.

To find peer‐reviewed published studies (published up through June 2019) that provided age‐specific survival rate data, we searched the Google Scholar database using the common and Latin names for each pinniped species and any and all combinations of the following search terms—“survival”, “survival rate”, “Cormack‐Jolly‐Seber”, “mark‐recapture”, “capture‐recapture”, and “mortality.” We used Google Scholar to maximize our opportunity to find usable studies, as it tends to return more results than other databases, such as Web of Science. We included survival data from all pinniped species for which we found reported survival or mortality data from nonlethal methods for at least one of the following stages: yearling (i.e., the survival interval between 1 year of age and 2 years of age), age at sexual maturity, and age at social maturity (Table 1). Studies varied in duration, models used to estimate survival (i.e., model type and its included covariates), methods of marking individuals, and sample size. However, these variations in survival rate estimation methods were unavoidable with available data, and we expect that these differences only accounted for minor changes in survival rate estimates. For studies using tagging techniques, many accounted for possible tag loss, but not all studies reported this adjustment to survival rate estimates. We limited our data to only studies that also included an uncertainty estimate associated with the estimated age‐ and sex‐specific survival rates (e.g., standard error or a confidence interval) to allow us to reflect this source of uncertainty in our analysis.

**TABLE 1:** Male survival rates and other life‐history traits of pinniped species

Species	Latitude and location name	Ages measured (years of age)	Time period	Survival rate (SE, 95% confidence interval) *N* Modeling method (closed or open population assumed) and covariates included	References
(a)					
Gray seal *Halichoerus grypus*	Sable Island, 43.93°	5–16	1969–1994	Sexual maturity: 0.98 (0.003, 35) Social maturity: 0.98 (0.003, 2,765) *N*_sexual_ = 18, *N_social_* = 48 *Jolly‐Seber (open), cohort*	Manske et al. (2002)
Harbor seal *Phoca vitulina*	Tugidak Island, 56.48°	1, 2–3, 4–7	2006–2011	Yearling: 0.78 (0.03, 0.71 – 0.84) Sexual maturity: 0.88 (0.03, 0.78 – 0.94) Social maturity: 0.88 (0.03, 0.78 – 0.94) *N*_marked as pups_ = 181 *Cormack*–*Jolly*–*Seber (closed)*	Hastings et al. (2012)
Weddell seal *Leptonychotes weddellii*	Erebus Bay, −77.7°	1, 2, 3, 4, 5, 6, 7, 8, 9, 10, 11, 12, 13, 14, 15, 16, 17, 18, 19, 20, 21, 22, 23, 24, 25, 26, 27, 28	1980–2015	Yearling: 0.60 (0.03, 0.51 – 0.75) Sexual maturity: 0.94 (0.02, 0.92 – 0.96) Social maturity: 0.93 (0.02, 0.91 – 0.94) *N*_yearling_ = 106, *N*_sexual_ = 101, *N*_social_ = 102 *Hierarchical model extensions of Cormack*–*Jolly*–*Seber in a Bayesian framework (closed), individual heterogeneity*	Brusa et al. (2020)
Southern elephant seal *Mirounga leonina*	Marion Island, −46.91°	1, 2, 3, 4, 5, 6, 7, 8, 9, 10, 11, 12	1983–1997	Yearling: 0.75 (0.051) Sexual maturity: 0.74 (0.062) Social maturity: 0.65 (0.128) *N*_marked as pups_ = 3,695 (assuming 50% sex ratio of marked individuals) *Cormack*–*Jolly*–*Seber (closed)*	Pistorius et al. (1999)
Northern elephant seal *Mirounga angustirostris*	Año Nuevo State Park, Santa Cruz, California, USA 37.11°	1, 2, 3, 4, 5, 6, 7, 8, 9, 10, 11, 12, 13, 14, 15	1985–2008	Yearling: 0.68 (0.62 – 0.74) Sexual maturity: 0.68 (0.62 – 0.75) Social maturity: 0.69 (0.53 – 0.82) *N*_yearling_ = 84, *N*_sexual_ = 17, *N*_social_ = 4 *Model extensions of Cormack*–*Jolly*–*Seber in a Bayesian framework (closed)*	Condit et al. (2014)
Mediterranean monk seal *Monachus monachus*	Cabo Blanco, Mauritania‐Morocco, 20.93°	4+	2004–2007	Social maturity: 0.87 (0.81–0.94) *N*_social_ = 57 *Jolly*–*Seber (open)*	Martinez‐Jauregui et al. (2012)
Hawaiian monk seal *Monachus schauinslandi*	French Frigate Shoals, 23.75°	1–2, 3–4, 5–17	1984–2003	Yearling: 0.56 (0.06, 0.43 – 0.68)^d^ Sexual maturity: 0.72 (0.09, 0.65 – 0.80)^d^ Social maturity: 0.89 (0.03, 0.83 – 0.93)^d^ *N*_marked as pups_ = 1,710 (assuming a 50% sex ratio of marked individuals) *Cormack*–*Jolly*–*Seber (closed)*	Baker & Thompson (2007)
Steller sea lion *Eumetopias jubatus*	Forrester Island, 54.8°	1, 2, 3, 4, 5, 6, 7, 8, 9, 10, 11, 12, 13, 14, 15, 16	2001–2009	Yearling: 0.69 (0.02, 0.65 – 0.72) Sexual maturity: 0.80 (0.02, 0.76 – 0.83) Social maturity: 0.73 (0.03, 0.68 – 0.78) *N*_marked as pups_ = 897 (assuming a 50% sex ratio of marked individuals) *N*_territorial_ = 53 *Cormack*–*Jolly*–*Seber (closed), multistate (closed), natal rookery, cohort, territoriality*	Hastings et al. (2018)
Australian sea lion *Neophoca cinerea*	Seal Bay, −35.98°	1.5–3, 3–14	1991–2002	Yearling: 0.88 (0.02, 0.83 – 0.92), Sexual maturity: 0.89 (0.03, 0.82 – 0.94) Social maturity: 0.89 (0.03, 0.82 – 0.94) *N*_marked as pups_ = 209 *Cormack*–*Jolly*–*Seber (closed), cohort*	McIntosh et al. (2013)
New Zealand sea lion *Phocarctos hookeri*	Sandy Bay, Enderby Island, −50.5°	1, 2, 3, 4–15	1989–2005	Yearling: 0.60 – 0.70, Sexual maturity: 0.98 (0.9 – 0.99) Social maturity 0.98 (0.9 – 0.99) *N*_marked as pups_ = mean of 845 per year (assuming a 50% sex ratio of marked individuals) *Multistate (closed), tag type*	Chilvers & MacKenzie (2010)
California sea lion *Zalophus californianus*	San Miguel Island, 34.03°	1, 2, 3, 4, 5, 6, 7, 8, 9, 10, 11, 12, 13, 14, 15, 16, 17, 18, 19	1987–2015	Yearling: 0.76 (0.71 – 0.80) Sexual maturity: 0.93 (0.91 – 0.94) Social maturity: 0.89 (0.89 – 0.87) *N*_yearling_ = 1,047, *N*_sexual_ = 613 *Burnham extension of Cormack*–*Jolly*–*Seber (closed), sea surface temperature, yearling weight, disease*	DeLong et al. (2017)
New Zealand fur seal *Arctocephalus forsteri*	Cape Gantheaume, Kangaroo Island, −36.07°	7–15	1992–1998	Social maturity: 0.76 (0.66 – 0.82) *N*_social_ = 34 *Kaplan*–*Meier estimate (closed), mass*	Troy et al. (1999)
Subantarctic fur seal *Arctocephalus tropicalis*	Amsterdam Island, −37.9°	pup, 1–2, 3–5	1995–2004	Yearling: 0.61 Sexual maturity: 0.96 (0.02)^a^ *N*_marked as pups_ = 365 *Cormack*–*Jolly*–*Seber (closed), cohort*	Beauple et al. (2005)

Species	Standard length in cm (SE, *N*)	Baculum size at maturity in cm (*SD*, *N*)	Mating strategy	Age at sexual maturity (years)	Age at social maturity (years)	References
(b)						
Gray seal *Halichoerus grypus*	230.7 (3.65, 244)	14.8 (2.36, 96)	Mild polygyny, aquatic and terrestrial dominance hierarchies, female defense	5	6	Hewer (1964)^c^, McLaren (1993)^b^, Hammill & Gosselin (1995)^c^, Twiss et al. (1998)^c^, Wilmer et al. (1999)^c^
Harbor seal *Phoca vitulina*	157.5 (1.42, 262)	13.7	Mild polygyny, aquatic and terrestrial lekking, display behavior	6	6	Scheffer & Kenyon (1963)^c^, McLaren (1993)^b^, Coltman et al. (1998)^c^, Boness et al. (2006)^c^, Hammill et al. (2010)^c^
Weddell seal *Leptonychotes weddellii*	239.7 (3.90, 38)	19.8 (1)	Moderate polygyny, aquatic resource defense, and display	3	5–11	Bartsh et al. (1992)^c^, McLaren (1993)^b^, Gelatt (2001)^c^, Morejohn (2001)^c^, Harcourt et al. (2007)^c^
Southern elephant seal *Mirounga leonina*	540.9 (38.8, 139)	33.10	Extreme polygyny, terrestrial female defense	3	9–10	Laws (1956)^c^, Stirling (1983)^c^, McLaren (1993)^b^, Galimberti et al. (2007)^c^
Northern elephant seal *Mirounga angustirostris*	448.6 (14.64, 162)	27.4 (1)	Extreme polygyny, terrestrial female defense	5	9–10	Scheffer & Kenyon (1963)^c^, Le Boeuf (1974)^c^, Stirling (1983)^c^, McLaren (1993)^b^
Mediterranean monk seal *Monachus monachus*	251.9 (11.1, 37)	21.5	Mild polygyny, aquatic resource defense	4	7	Stirling (1983)^c^, Samaranch & González (2000)^b^, Gucu et al. (2004)^c^, Pastor et al. (2011)^c^, Fitzpatrick et al. (2012)^c^, Murphy et al. (2012)^c^
Hawaiian monk seal *Monachus schauinslandi*	198.4 (2.30, 51)	18.3 (1)	Seasonal monogamy, aquatic female defense	4	8–9	Kenyon & Rice (1959)^c^, Scheffer & Kenyon (1963)^c^, Stirling (1983)^c^, Reif et al. (2004)^b^, Harting et al. (2007)^c^
Steller sea lion *Eumetopias jubatus*	310.2 (1.85, 101)	18.1 (1.02, 22)	Extreme polygyny, terrestrial resource defense	3	9–13	Pitcher & Calkins (1981)^c^, McLaren (1993)^b^, Miller et al. (2000)^c^, Parker & Maniscalco (2014)^c^
Australian sea lion *Neophoca cinerea*	192.2 (0.25, 35)	NA	Moderate polygyny, terrestrial female defense	3	3	McIntosh (2007)^b^, Higgins (1990)^c^, McIntosh (2007)^b^
New Zealand sea lion *Phocarctos hookeri*	220 (1.7, 20)	17.5 (2.59)	Moderate polygyny, terrestrial resource defense	4	7–9	Bartholomew (1970)^c^, McConkey et al. (2002)^b^, Campbell et al. (2006)^c^, Robertson et al. (2006)^c^, Duignan & Jones (2007)^c^, Childerhouse et al. (2010)^b^, Foote (2018)^c^
California sea lion *Zalophus californianus*	229.8 (4.4, 44)	12.1	Mild polygyny, terrestrial resource defense	5	9–10	Scheffer & Kenyon (1963)^c^, Peterson & Bartholomew (1967)^c^, McLaren (1993)^b^, Aurioles‐Gamboa & Zavala‐González (1994)^c^, Flatz et al. (2012)^c^
New Zealand fur seal *Arctocephalus forsteri*	144 (2.3, 39)	9.6 (0.08, 2)	Moderate polygyny, terrestrial resource defense	4	7	Mattlin (1978)^c^, Carey (1991)^c^, Troy et al. (1999)^c^, Dickie & Dawson (2003)^b^, Duignan & Jones (2007)^c^, McKenzie et al. (2007)^c^
Subantarctic fur seal *Arctocephalus tropicalis*	163.7 (5.8)	9.5 (123)	Mild polygyny, terrestrial female defense	4	8–9	Bester (1990)^c^, Bester & Jaarsveld (1994)^b^
Harp seal, *Pagophilus groenlandicus*	172.2	17.4 (1.44, 706)	Mild polygyny, aquatic social groups	4	4	Sergeant (1976)^c^, Merdsoy et al. (1978)^c^, Miller et al. (1998)^c^, Nordøy et al. (2008)^b^
Leopard seal *Hydrurga leptonyx*	294 (19.0, 44)	23.3 (2)	Mild polygyny, ice	4	4	Scheffer & Kenyon (1963)^c^, Stirling (1983)^c^, Rogers (2003)^c^, Rogers (2007)^c^, Gray et al. (2009)^b^
Crabeater seal *Lobodon carcinophaga*	225.4 (1.39, 51)	22.0 (1)	Seasonal monogamy, defense of single female on ice	3	5	Scheffer & Kenyon (1963)^c^, Siniff et al. (1979)^c^, Bengtson & Siniff (1981)^c^, McLaren (1993)^b^
South American sea lion *Otaria flavescens*	256.5 (3)	NA	Moderate polygyny, terrestrial resource defense	4	9	Campagna & Boeuf (1988)^c^, Rosas et al. (1993)^b^, Grandi et al. (2010)^c^
Galapagos sea lion *Zalophus wollebaeki*	205	NA	Mild polygyny, semi‐aquatic display	4	5	Pörschmann et al. (2010)^c^, Trillmich et al. (2014)^b^, Kalberer et al. (2018)^c^
Galapagos fur seal *Arctocephalus galapagoensis*	152	NA	Moderate polygyny, terrestrial resource defense	3	8–10	Trillmich (1984)^b^, Trillmich (1987)^c^
Antarctic fur seal *Arctocephalus gazella*	212.2 (13.7, 78)	11.3	Moderate polygyny, terrestrial resource defense	3	8	Bonner (1958)^c^, Rand (1967)^c^, Payne (1979)^c^, McLaren (1993)^b^, Hoffman et al. (2003)^c^, Fitzpatrick et al. (2012)^c^
Cape fur seal *Arctocephalus pusillus*	220	12.0 (0.8, 20)	Moderate polygyny, terrestrial female defense	3	9–10	Stirling (1983)^c^, Rand (1955)^c^, Oosthuizen & Miller (2000)^c^, Arnould & Warneke (2002)^b^
Northern fur seal *Callorhinus ursinus*	189.3 (2.59, 291)	13.0 (0.6, 4)	Extreme polygyny, terrestrial female defense	3	7–10	Scheffer (1950)^c^, Bartholomew & Hoel (1953)^c^, Johnson (1968)^c^, York (1983)^c^, McLaren (1993)^b^
Pacific walrus *Odobenus rosmarus*	310.1 (6.9, 168)	NA	Moderate polygyny, terrestrial female defense	8	15	Fay (1982)^c^, McLaren (1993)^b^, Sjare & Stirling (1996)^c^

Note

Male‐specific survival data from live, free‐ranging animals were available for 13 pinniped species (a). Survival rates are provided for as many ages of each species as were made available from studies with empirical data for annual survival rates. Covariates reported here are in addition to age and time that were used in the presented estimated survival rates. Additional life‐history trait data are presented for 23 pinniped species (b). The standard length of a species indicates the mean or asymptotic length of an adult. All estimates of life‐history traits are single estimates from one representative publication for the species; in many cases, the information was only available from a single publication. In instances when multiple studies were found that provided information about the same life‐history trait, the study that met all model assumptions and had the greatest sample size was selected. Standard errors/standard deviations and sample sizes are presented when available.

^a^ Survival estimates from pooled sexes, as no significant difference in survival was found between sexes

^b^ Reference for standard length estimates, means from multiple populations provided for some species when available

^c^ Reference for mating strategy.

^d^ Age‐specific estimates across all years of study were only presented in graphical format, and point estimate values were provided for each individual year of the study. We selected a year with point estimates that was representative of the average of estimates across all years that were presented in graphical format.

For each species, we also searched the literature for species‐specific information on body size, age at sexual maturity and at social maturity, type of male–male contest, baculum length, and specific type of mating system (Table 1). For most species, data for each of the life‐history traits were compiled from different populations (i.e., from different studies), but data for multiple life‐history traits from the same population (study) were used whenever possible. We summarized quantitative life‐history traits using averages and by sex when appropriate, including an associated uncertainty measurement (e.g., a standard error for an average) when possible.

We categorized our data collection into two main tiers of age‐specific annual survival rates based on available information for each species. The first tier, which includes those species for which we have the most complete data, included age‐specific survival with associated measurement error from all ages throughout the known life span for each species from a single population. Some studies grouped multiple ages (e.g., 1 year of age, 2–3 years of age, 3–7 years of age), and we used these age groupings as our second tier of sex‐ and age‐specific data collection. Depending on available survival data for each age, species were included in the analyses of survival rates for the following ages: yearling, age at sexual maturity, and/or age at social maturity. When multiple papers reported survival rates for the same species, we selected papers that presented age‐specific results with the smallest age ranges (e.g., a paper with survival rate data for “2 to 3 and 4 to 7 years of age” was used instead one with survival rate data for “adults ≥3 years of age”). Given that survival rate estimates for some species included in our analyses were not specific to a single age but were estimated from an age range that included either the age at sexual maturity or the age at social maturity (tier 2 species), we assume that the survival rate estimated for the age range that includes sexual or social maturity is representative of the single age at sexual or social maturity. If multiple papers presented age‐specific survival rate data for each age, we selected the study that provided the most complete methods (e.g., accounting for individual heterogeneity to represent variation in unmodeled traits that could relate to survival rates). We used this method to maximize the comparability and generalizability of empirical data for our comparative analysis. For one of the second‐tier species (*Arctocephalus tropicalis*, age at sexual maturity), the authors reported that there was no significant difference in annual survival rates between males and females and presented survival rates from male and female grouped data.

### 2.4 Example with real pinniped data

We applied our model to available male pinniped data for survival rate at the age of social maturity and standard body length (*n* = 12). We ran an MCMC with 3 chains, a burn‐in of 10,000 iterations, and retained every 5th sample to reduce autocorrelation in the chains for a final sample of 300,000 MCMC iterations used to fit the model. We present 90% credible intervals for all analyses we conducted (90% CrI). When describing reported data from empirical studies, we present 95% confidence/credible intervals for survival rates provided by the empirical studies, as they provided 95% confidence/credible intervals but not enough essential information to transform these values to 90% confidence/credible intervals. As our model applied to real data results in more noise than signal (direct result of limited data availability/quality), we also present graphical analyses. Graphical analyses can be useful for discerning patterns and possible relationships between life‐history traits but should not be used for quantitative prediction (Roff, 1992). Although graphical analyses can be performed to evaluate the optimal combination of trait values to maximize fitness (Real & Ellner, 1992; Roff, 1992), we simply employ a visualization approach.

## 3 RESULTS

### 3.1 Simulation study

The 95% credible interval coverage across each of the model parameters demonstrated the ability of the model to capture data‐generating parameters with nominal coverage. The percentage of the simulated data means that were within the 95% credible intervals of the posterior predictive distribution ranged from 94% for $\sigma_{\varepsilon }$ to 100% for α (Table 2).

**TABLE 2:** Summary of results from 100 iterations through unbiased simulation study of our modeling approach

Parameter	Average estimate (data‐generating values)	Average 95% CrI width	Capture rate	Average effective sample size	Average $\hat{R}$	Average bias
*α*	0.22 (0.2)	0.80	1.00	4,871	1.001	0.023
*β*	−0.99 (−1)	0.99	0.97	2,212	1.002	0.011
*σ_ε_*	0.50 (0.5)	0.53	0.94	817.2	1.004	−0.002

Note

The capture rate for each parameter represents the percentage of simulated datasets that, after fitting the model, resulted in 95% posterior intervals including the true data‐generating value used for the parameter.

### 3.2 Example with standard body length data

**FIGURE 1:**
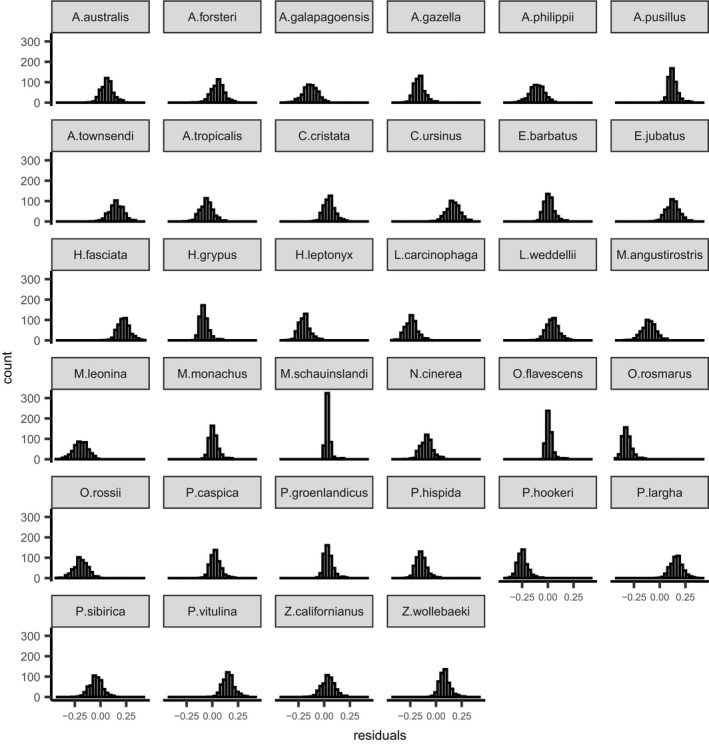
Residuals by species for simulated data. Residuals were calculated from subtracting the predicted log‐odds of survival at the age of social maturity from the observed log‐odds of survival at the age of social maturity (generated from our simulated datasets). The predicted log‐odds of survival values were simulated from the posterior distribution of the Bayesian phylogenetically controlled generalized least squares model

**FIGURE 2:**
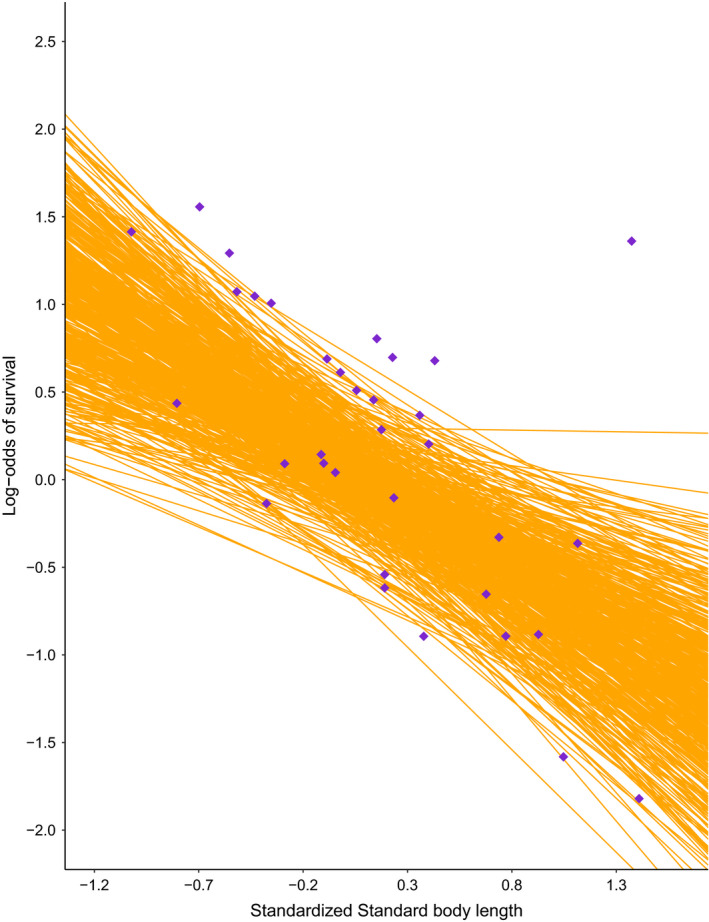
Posterior predictive check comparing model estimates and simulated data. The relationship between standard body length and the log‐odds of survival for male pinnipeds at the age of social maturity is shown for the observed data generated from our simulated datasets (purple diamonds) and simulated data from the Bayesian phylogenetically controlled generalized least squares posterior distribution (orange regression lines)

Visual inspection of model diagnostics revealed abundant mixing of chains and all $\hat{R}$ values lower than 1.1, which indicated model convergence (Gelman, 2013). Our posterior predictive checks demonstrated the lack of statistical power with small sample sizes in that uncertainty associated with point estimates was large, but our goodness‐of‐fit testing did not reveal any obvious issues using simulated data (Figures 1 and 2). Given the complexity of the model and possible low sample size with future applications, model convergence can be slow (specifically, autocorrelation in chains can be high). We recommend careful selection of priors and attention to possible effects of prior when sample sizes are moderate. From the results of applying our model to real data, the estimated change in the log‐odds of survival for one‐unit increase in standard body length was −0.82 (90% CrI = −2.07, 0.49). A negative value would indicate that larger body sizes are related to lower survival rates, but a credible interval overlapping zero indicates that an increase in body size could result in higher or lower survival rates and provides further evidence of the necessity to increase the number of species with male survival rate data. Additionally, we observed large uncertainty in the posterior for λ (mean = 0.92, 90% CrI = 0.80, 0.99), which is to be expected with a sample size of fewer than 20 species (Münkemüller et al., 2013) and can influence the relationship between covariates analyzed and the response variable (Chamberlain et al., 2012).

### 3.3 Summary of survival rate data for male pinnipeds

**FIGURE 3:**
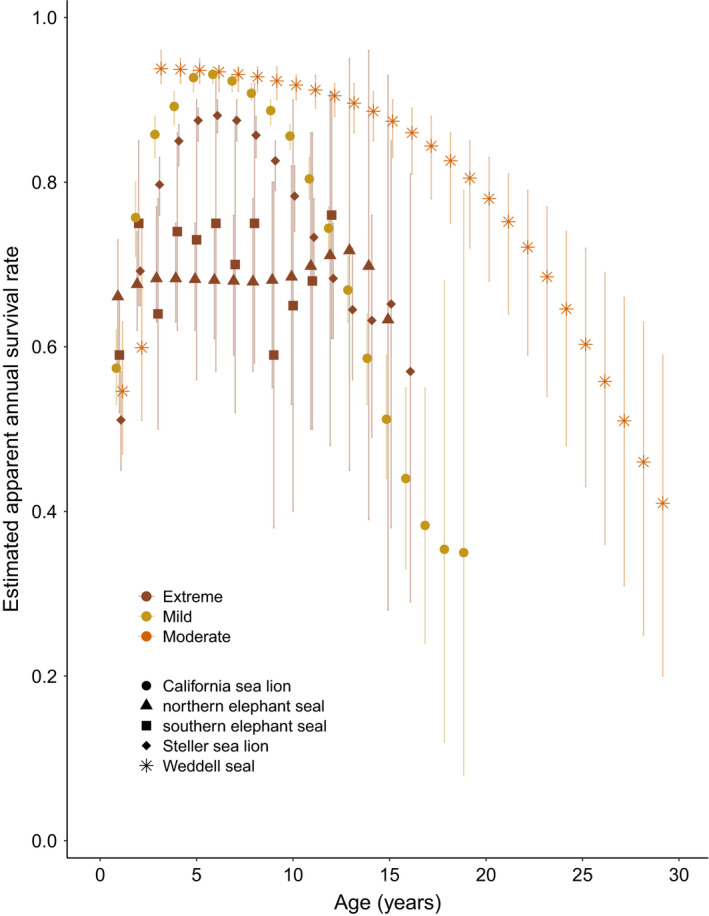
Age‐specific male survival rates for 5 pinniped species. Error bars indicate either 95% confidence intervals or 95% credible intervals. Data are displayed according to species (symbols) and mating systems of extreme, moderate, and mild polygyny (colors)

**FIGURE 4:**
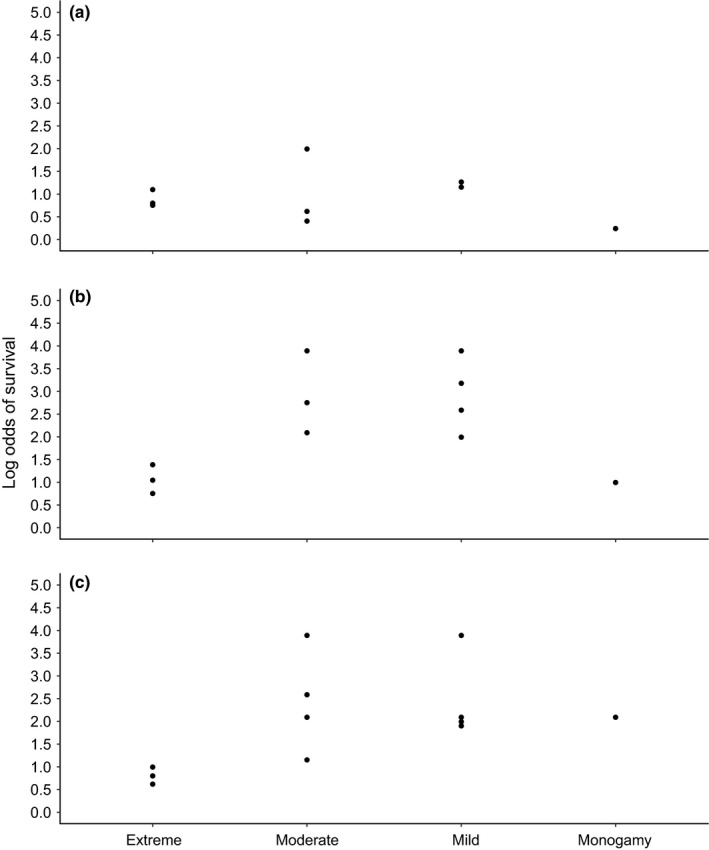
Empirical relationship between mating system, which is defined using the guidelines of Le Boeuf (1991), and interspecific variation in survival rates for pinnipeds at 2 years of age (a), age at sexual maturity (b), and age at social maturity (c). Survival rates are on the logit scale

We were able to compile survival data for 13 species (Table 1) and additional life‐history trait data for 23 species (Table 1). Sequence data availability also varied across species, as some were heavily represented, but 8 extant pinniped species had no sequence information available from the NCBI nucleotide database. We found complete age‐specific male survival data for five species (first tier, see Methods) and data for an additional eight species for which some ages were combined (second tier). Similar to many other life‐history traits, survival rates of male pinnipeds vary substantially across species. Of the five species with full age‐specific male survival estimates, peak survival rates ranged from 0.72 (95% CI = 0.45, 0.95, Condit et al., 2014) for northern elephant seals to 0.94 (95% CrI = 0.92, 0.96, Brusa et al., 2020) for Weddell seals (Figure 3). However, the age at which peak survival rates were reached varied considerably among species, and uncertainty associated with age‐specific estimates also varied among species. Although we did not have a strong enough signal in our limited subset of reliable data for pinniped species to assess a relationship using our modeling approach, we see a weak log‐linear relationship between mating system and survival rate in male pinnipeds in our data visualizations/graphical analyses (Figures  4).

## 4 DISCUSSION

Comparative studies are a useful method for understanding life‐history trait evolution (e.g., Dines et al., 2015; Festa‐Bianchet, 2012; Fitzpatrick et al., 2012; Lemaître et al., 2020; Tidière et al., 2015). However, robust comparative analyses present several challenges, such as incorporating uncertainty. We have built upon previous work from de Villemereuil et al. (2012) and Ferrari and Cribari‐Neto (2004) to present a versatile Bayesian model that can accommodate a response variable measured on the probability scale and can incorporate measurement error for response variables, covariates, and phylogenetic tree topologies and branch lengths. Although comparative analyses provide an opportunity to make higher‐order conclusions than afforded by the individual empirical studies upon which they are based (Arnqvist & Wooster, 1995), they require assumptions that can be unrealistic. For example, it can be difficult to acquire comparable data for multiple characteristics of a set of species that represent a taxonomic group (Freckleton, 2009; Gerstner et al., 2017). Additionally, focusing on a single taxonomic group can improve the comparability of data for a comparative analysis (Lanyon, 1994), but narrowing the scope of inference to a single monophyletic group can reduce sample size. We recommend the careful consideration of inclusion criteria for life‐history trait data in future comparative studies even though it can limit sample sizes. From our exploration of life‐history traits in male pinnipeds, mating system appears to be related to interspecific variation in annual survival rates of male pinnipeds for each stage. However, additional high‐quality survival rate data are necessary for further exploration of this possible connection. As age‐ and stage‐specific survival rates of male pinnipeds varied widely across species, pinnipeds should serve as a useful taxonomic group for investigating broad questions related to life‐history trait trade‐offs.

We posit that the most critical step of a comparative study is selecting which empirical data to include in the analysis. As demonstrated here with survival rates of male pinnipeds, the use of strict selection criteria can severely limit sample size. However, one must carefully consider the consequences of including additional data. For example, survival rate data are often analyzed differently for adult male and female pinnipeds. Because it is often obvious to identify which females have bred in a given year (i.e., they are found with a pup), female survival rates are sometimes presented as subgroups consisting of prebreeders, breeders, or nonbreeders (e.g., Beauplet et al., 2006; Paterson et al., 2018). Survival rate data reported for subgroups would not be comparable to data that included an unbiased sampling of the population. For this reason, data about males, rather than females, might be more useful for comparative analyses.

Sexual selection is a common focus for comparative studies (e.g., Dines et al., 2015; Fitzpatrick et al., 2012; Lüpold et al., 2014; Promislow, 1992), but only a few studies have investigated the relationship between survival rates and sexually selected traits (Festa‐Bianchet, 2012; Kemp, 2006). Strong competition for mating opportunities often constrains male life histories, especially in species with polygynous mating systems (Festa‐Bianchet, 2012; Mysterud et al., 2003). We found that pinniped species exhibiting extreme polygyny tended to have the lowest adult survival rates. For nearly all species evaluated within all of the literature we considered, the estimated survival rate at the age of sexual (*n* = 7) and social maturity (*n* = 8) exceeded the estimated survival rate for yearlings. However, yearlings had higher survival rate estimates than adults reaching sexual or social maturity in southern elephant seals, a species that engages in extreme polygyny. Pistorius et al., (1999) suggested that the relatively low adult survival rate estimate for male southern elephant seals might be the result of stress from male–male contests and the inability of males to meet increased nutritional demands coincident with the breeding season and growth spurts following sexual maturity.

To fully address broadscale life‐history trait evolution questions, robust empirical data are necessary. This study specifically underscores the paucity of data on male survival rates for many pinniped species and the importance of high‐quality and long‐term data collection for survival rates. High‐quality survival data for many long‐lived vertebrate species are absent because many logistical and temporal challenges often accompany the collection of survival data for these species (Gaillard et al., 1993; Hiby & Mullen, 1980; Lebreton et al., 1992, 1993). Wickens and York (1997) initially brought this issue to attention for pinniped species, specifically for fur seals, and recent studies focusing on survival rates in pinnipeds have emerged. To increase the abundance of high‐quality data for comparative studies of survival rates in pinniped species, data from live animals, such as mark–recapture or telemetry methods, are necessary (Loison et al., 1999). There is high potential for increasing the number of demographic studies on additional pinniped species because their amphibious behavior, annual reproductive pulse found in most species, and predictable aggregation patterns facilitate mark–recapture techniques.

Although data deficiencies prevent a full analysis of the relationship between survival rates and other life‐history traits in male pinnipeds, our model is flexible and broadly applicable for other taxa. As multiple life‐history traits are measured or summarized on the probability scale (e.g., survival, reproduction rates), but few comparative studies have investigated such life‐history traits, we demonstrate a widely applicable model for such analyses. Further, we have identified a possible link between mating system and survival rate in male pinnipeds that warrants further exploration. We exemplify the importance and relative ease of incorporating uncertainty into a comparative analysis using a Bayesian model when empirical data are presented with estimates of uncertainty, and the model can be easily adjusted to analyze relationships between life‐history traits in other species groups. Our comparative study illustrates the importance of high‐quality survival estimates, and advances in quantitative population biology have provided avenues for acquiring these estimates (Clutton‐Brock & Sheldon, 2010). We provide a foundation for understanding the complexities of the relationships between survival rates and other life‐history traits in male pinnipeds. Our initial exploration allowed us to make novel predictions about life‐history strategies in male pinnipeds, and, to provide more concrete answers to our research questions posed in the Introduction, we plan to follow up with a more formal analysis once data for additional species are available. For taxa that are not restricted by a dearth of data, we provide a basic model that can be extended as a flexible way forward including an example of its use with simulated data and our limited data for male pinnipeds.

## CONFLICT OF INTEREST

We declare that we do not have any conflict of interest.

## AUTHOR CONTRIBUTIONS

**Jamie Louise Brusa:** Conceptualization (lead); Data curation (lead); Formal analysis (equal); Investigation (equal); Methodology (equal); Project administration (lead); Validation (equal); Visualization (equal); Writing‐original draft (lead); Writing‐review & editing (equal). **Jay J Rotella:** Conceptualization (supporting); Data curation (supporting); Formal analysis (equal); Funding acquisition (lead); Investigation (equal); Methodology (equal); Validation (equal); Visualization (equal); Writing‐review & editing (equal). **Katharine Michelle Banner:** Conceptualization (supporting); Data curation (supporting); Formal analysis (equal); Investigation (equal); Methodology (equal); Validation (equal); Visualization (equal); Writing‐review & editing (equal). **Patrick Ross Hutchins:** Conceptualization (supporting); Data curation (supporting); Formal analysis (equal); Investigation (equal); Methodology (equal); Validation (equal); Visualization (equal); Writing‐review & editing (equal).

## Supporting information

Supplementary Material

Table S1

Supplementary Material

Supplementary Material

## Data Availability

The data presented in this manuscript can be found using the following DOI accession number: 10.5061/dryad.kd51c5b5h. The sequence data for phylogenetic analyses were taken from NCBI (accession numbers are presented in Table S1), and sources for all life‐history trait data are presented in Table 1.
