# Supplementary material for: Challenges and opportunities for comparative studies of survival rates: An example with male pinnipeds
Source: Ecol Evol. 2021 May 8;11(12):7980–99. doi: 10.1002/ece3.7627 (PMC8216918; doi:10.1002/ece3.7627)
Supplement: Supplementary file 1 — Supplementary Material [file ECE3-11-7980-s002.pdf]

Supplementary Materials for *Challenges and opportunities for comparative studies of survival rates: an example with male pinnipeds*

Jamie L. Brusa, Jay J. Rotella, Katharine M. Banner, Patrick R. Hutchins

Bayesian Measurement Error model with Phylogenetic Structure and Uncertainty

The following is our example code for demonstrating the functionality of our Bayesian model that incorporates uncertainty in the response variable (survival rate), covariate (standard body length), and phylogeny using an unbiased simulation study. We also provide a realistic example for pinnipeds with our small dataset. To incorporate phylogenetic similarities among species, the inverse of the standardized phylogenetic tree can be used as the true correlation structure among observations in a Bayesian version of phylogenetic generalized least squares. Phylogenetic uncertainty can also be accounted for by sampling from many candidate trees (described in deVillemereuil et al. 2012).

Accomodating measurement error on the probability scale Measurement error variances need to either be assumed to be known for the explanatory and response variables in this modeling framework, or there needs to be multiple observations for a species for measurement error variances to be estimated from the data. In our example, we have a probability as the response variable and an X covariate that can be assumed to have normally distributed measurement error.

To address a response variable on the probability scale, we assume a beta measurement-error (ME) distribution for the observed survival rates conditional on the true survival rates. We use the regression parameterization of the beta distribution to allow the ME distribution to be centered on the true survival probability with standard deviation close to the observed standard error. To do this, we used the observed survival rates and their associated standard errors on the probability scale. This has a bit of an Empirical Bayesian approach, as we are using the observed data to help set a prior for the survival rates for each species. We wrote a function in R (R Core Team 2019), `beta_loss` to search through a grid of α and β values (i.e., hyper-parameter values for our measurement error distribution on survival rates) such that the mean and variance of the beta distribution were as close as possible to the observed values. It is important to note that the range of values for α and β that are used in the `beta_loss` function will affect how informative this distribution is — generally, a larger maximum will translate to a more informative prior distribution on survival rates.

In this case, we chose the upper endpoint of 10 because we were treating survival rate as a continuous measure in our model. If we would have had access to the raw data going into the survival probabilities, we would want to think about setting this value with respect to the maximum number of additional successes and failures we felt comfortable adding to the data [Gelman et al. (2014); pg. 34-35]. The maximum values for α and β in the `beta_loss` function should be set with the specific problem in mind; they can be modified by adjusting the call to the `seq` function in the `test_grid` object within the `beta_loss` function.

```r
beta_loss <- function(mean = 0.86, se = 0.01, disp = TRUE){
  test_grid <- expand.grid(alpha = seq(0.01, 10, by = 0.1),
                           beta = seq(0.01, 10, by = 0.1))
  alpha <- seq(0.01, 10, by = 0.1)
  beta <- alpha
  # E(Y) = alpha/(alpha + beta)
  e_y <- test_grid[,1]/(test_grid[,1] + test_grid[,2])
  mean_loss <- abs(mean - e_y)
  # Var(Y) = alpha*beta/((alpha + beta)^2(alpha + beta + 1))
  var <- test_grid[,1]*test_grid[,2]/((test_grid[,1] +
                                        test_grid[,2])^2*(test_grid[,1] +
                                                          test_grid[,2] + 1))
  # calculate total abs loss for different alpha, beta combos
  tot_loss <- abs(mean - e_y) + abs(se^2 - var)
  # find min
  out <- test_grid[which(tot_loss == min(tot_loss)),]
  # inform prior, if command to show plot and output for loss if desired
  if(disp == TRUE){
    curve(dbeta(x, round(out[1,1], 1), round(out[1,2], 1)), ylab ="density",
          xlab = "X", main = paste("X ~ Beta(", round(out[1,1],1),
                                   ",", round(out[1,2], 1), ")"))
    print(paste("total absolute loss = ",
                round(tot_loss[which(tot_loss == min(tot_loss))], 6)))
  }
  return(out)
}

# To find informative priors for all species, apply the beta loss function
# to survival rate estimates and associated standard errors for all species
```

The following illustrates an example of the beta loss function using one species. For this particular species, we wanted the measurement error distribution to be centered around 0.6 with a standard deviation of 0.03 to reflect the standard error of that survival rate probability. The `beta_loss` function displays a plot of the "best" (according to custom loss function) beta distribution, its parameter values, and the total absolute loss (the smaller the "better" with respect to achieving the desired mean and variance of the beta distribution).

```r
beta_loss(mean = .6, se = 0.03)
```

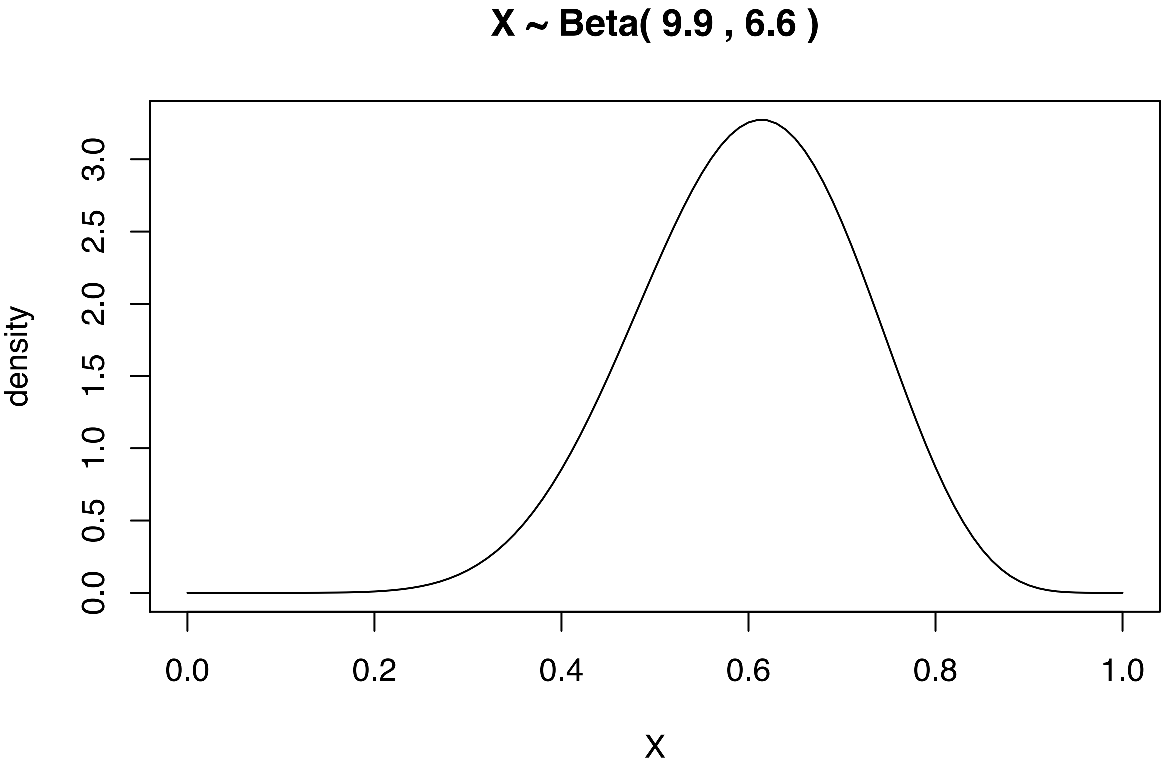


```
## [1] "total absolute loss =  0.012921"

##      alpha beta
## 6700  9.91 6.61
```

Simulation study

Below, we have detailed our simulation study that was motivated by the hypothetical situation in which we have data for the 34 extant pinniped species. Our data simulation function generates survival rate estimates and standard body length estimates that are observed with known measurement error. That is, standard errors for these values are assumed to be known and were engineered to be similar to those observed in the real data. The same measurement error values are used for all data sets in the simulation study.

Data generating function The following code takes user-specified data-generating values for all parameters and generates observed survival probabilities and standard body lengths that are measured with error. The data are generated assuming a logit-linear relationship between median survival probability and standard body length. In this example, the latent structure is assumed to be: $X_i \sim N(\mu_x = 0, \sigma_x = 0.5), i = 1, 2, ..., 34$; $\mathbf{Y} = 0.2 - 2\mathbf{X} + \boldsymbol{\epsilon}$, where $\boldsymbol{\epsilon} = MVN(\mathbf{0}, 0.55^2\boldsymbol{\Sigma})$, and $\mathbf{p} = logit^{-1}(\mathbf{Y})$). The observed variables are generated conditional on the latent variables according to: $W_i|X_i \sim N(X_i, \sigma_{ui}^2)$ and $D_i|p_i \sim Beta(p_i\phi, p_i(1-p_i)\phi)$, where σ_{ui} and ϕ are also observed as data (ϕ is derived from the observed estimate and SE for the survival rate as described previously (using the `beta_loss` function)).

```r
# Simulation code (simplified version using a single,
# simulated tree for all 34 extant species)
library(rjags)
```

```
## Loading required package: coda
```

```
## Linked to JAGS 4.3.0
```

```
## Loaded modules: basemod,bugs
```

```r
library(runjags)
library(coda)

# set up phylogenetic correlation structure
# change working directory to the path where the consensus tree is stored
# (simumat.csv is in Desktop/Complete models on this machine)
setwd("~/Desktop/Complete models")
phylo <- read.csv("simumat.csv", row.names = 1)
phylo <- as.matrix(phylo, nrow = dim(phylo)[1], ncol = dim(phylo)[2])
phylo_scaled <- phylo/max(diag(phylo))
inv_phylo <- solve(as.matrix(phylo_scaled))

# generate common measurement error variance for the body lengths
# and survival probabilities for the simulation study
set.seed(22221)
sigma_u <- 0.1*abs(rnorm(34))
se_surv <- runif(34, 0.01, 0.03)
se_surv <- matrix(t(as.data.frame(se_surv)), nrow = 34,
                  ncol = 1, byrow = TRUE)

# Data-generating function - generates one fake dataset
data_gen_full <- function(alpha = 0.2, beta = -1, n_spp = 34,
                          mu_x = 0, sigma_x = 0.5, sigma_eps = 0.5,
                          sigma_u, se_surv,
                          inv_phylo){
  x <- rnorm(n_spp, mean = mu_x, sd = sigma_x)
  Sig <- sigma_eps^2*solve(inv_phylo)
  y <- alpha + beta*x + MASS::mvrnorm(1, mu = rep(0,n_spp), Sigma = Sig)
  p <- faraway::ilogit(y)
  w <- rnorm(n_spp, mean = x, sd = sigma_u)
  surv_dat <- cbind(p, se_surv)
  ab_surv <- apply(surv_dat, 1, function(x){
    beta_loss(mean = x[1], se = x[2], disp = FALSE)
  })
  ab_surv <- do.call(rbind, ab_surv)
  phi <- apply(ab_surv, 1, sum)
```

```
  d <- rep(NA, n_spp)
  d2 <- rep(NA, n_spp)
  for(i in 1:n_spp){
    d[i] <- rbeta(1, shape1 = ab_surv[i,1], shape2 = ab_surv[i,2])
  }
  df <- data.frame(D = faraway::logit(d), W = w, phi = phi)
  return(df)
}
```

Simulation wrapper The simulation wrapper allows the user to iterate a user-specified number of times through the following steps:

- Generate a single realization from the data-generating process described above
- Fit the Bayesian model described in the simulation study section of the manuscript
- Store posterior samples and summary results in a list
- Repeat the process for the desired number of iterations

We ran a simulation with 100 iterations of this process, which took about 16.1 hours to complete. Before running the simulation, we tuned the MCMC by visually assessing trace plots with 3 chains, posterior density plots, and other MCMC diagnostics (e.g., $\hat{R}$ and n_{eff}). High autocorrelation in the MCMC required a burnin = 15000, sample = 35000, and thin = 4 before convergence was indicated. Coverage for each of the parameters of interest ranged from 94% for σ_ϵ to 100% for α (from 100 iterations).

```
# simulation wrapper function
bayes_me_pXY <- function(n_iter, alpha = 0.2, beta = -1, n_spp = 34, se_surv,
                         inv_phylo, mu_x = 0, sigma_x = 0.5, sigma_eps = 0.5,
                         n.chains = 3, sigma_u, adapt = 5000,thin = 4,
                         burnin = 15000,  sample = 35000
                           ){
  slr_summ <- as.list(1:n_iter)
  computation_time <- as.list(1:n_iter)
  mcmc_results <- as.list(1:n_iter)
  df_list <- as.list(1:n_iter)

  # set progress bar
  pb <- txtProgressBar(min = 0, max = n_iter, style = 3)

  for(i in 1:n_iter) {
    df <- data_gen_full(alpha = alpha,
                        beta = beta,
                        sigma_u = sigma_u,
                        se_surv = se_surv,
                        sigma_eps = sigma_eps,
                        inv_phylo = inv_phylo,
```

```
                        n_spp = n_spp,
                        mu_x = mu_x, sigma_x = sigma_x)
W <- df$W
D <- faraway::ilogit(df$D)
phi <- df$phi
params_me_phylo <- c("alpha", "beta", "sigma_eps")
data_me_phylo <- list(Nspec = n_spp,
                      W = W,
                      D = D, #on prob scale
                      sigma_u = sigma_u,
                      inv_phylo = inv_phylo,
                      phi = phi)
inits <- function(){list(alpha = runif(1,0,1),
                         beta = runif(1,-1.5,-0.5))}

bayes_me_phyloXY <- "model {

Y[1:Nspec] ~ dmnorm(meanY[], TAUY[,])
X[1:Nspec] ~ dmnorm(meanX[], TAUX[,])

for(i in 1:Nspec) {

meanY[i] <- alpha + beta * X[i]

W[i] ~ dnorm(X[i], tau_u[i])
tau_u[i] <- pow(sigma_u[i], -2)

meanX[i] ~ dnorm(mu_x, tau_x)

p[i] <- ilogit(Y[i])

D[i] ~ dbeta(p[i]*phi[i], (1-p[i])*phi[i])
}

# phylo strucure
TAUY <- tau_eps*inv_phylo
TAUX <- tau_x*inv_phylo

# priors
tau_eps ~ dgamma(0.1,0.1)
sigma_eps <- 1/sqrt(tau_eps)
mu_x  <- 0
sigma_x <- 0.5
tau_x <- 1/(sigma_x*sigma_x)
```

```
    alpha ~ dnorm(0,0.25)
    beta ~ dnorm(0,0.25)
    }"

    me_phylo <- run.jags(model = bayes_me_phyloXY,
                         monitor = params_me_phylo,
                         data = data_me_phylo,
                         n.chains = n.chains,
                         adapt = adapt,
                         thin = thin,
                         burnin = burnin,
                         sample = sample, inits = inits)

# store summary output for iteration i in list
    post_summ <- as.data.frame(summary(me_phylo))

    # compare posterior summaries to data generating values to track
    #coverage
    post_summ$iter <- i
    post_summ$par <- c(rownames(post_summ))

    dg_values <- c(alpha, beta, sigma_eps)

    post_summ$capture <- as.numeric(dg_values >= post_summ$Lower95 &
                                    dg_values <= post_summ$Upper95)
    post_summ$cri95_width <- post_summ$Upper95 - post_summ$Lower95

    #save the summary output from this realization in the list
    slr_summ[[i]] <- post_summ
    mcmc_results[[i]] <- me_phylo$mcmc
    df_list[[i]] <- df
    setTxtProgressBar(pb, i)
    print(paste("Completed iteration", i, "of", n_iter))
  }
  close(pb)

  # return the list of summary output from each run of the JAGS model
  jags_summ <- do.call(rbind, slr_summ)
  out <- list(jags_summ = jags_summ, mcmc_results = mcmc_results,
              df_list = df_list)
}

sim_me_phyloXY <- bayes_me_pXY(n_iter = 100, se_surv = se_surv,
                               adapt = 5000, inv_phylo = inv_phylo,
```

```
                                sigma_u = sigma_u, thin = 4,
                                burnin = 15000,  sample = 35000)

# summarize simulation results
library(tidyverse)
sim_phyloXY <- sim_me_phyloXY$jags_summ %>%
  group_by(par) %>%
  summarise(cap_rate = mean(capture),
            avg_est = mean(Mean),
            ci_width = mean(Upper95 - Lower95),
            avg_L95 = mean(Lower95),
            avg_U95 = mean(Upper95),
            avg_rhat = mean(psrf),
            avg_neff = mean(SSeff))

# recreate table in manuscript
sim_phyloXY$dg_values <- c(0.2, -1, 0.5)
sim_phyloXY$avg_bias <- sim_phyloXY$avg_est - sim_phyloXY$dg_values
pander::pander(sim_phyloXY, caption = "simualtion results ME on X and Y,
               phylo structure on logit-linear correlation strucutre and X")
```

Here, we show trace plots and density plots for one iteration of the simulation (code was not echoed, but is available in the .Rmd for running one iteration of the simulation).

```
#Evaluate model convergence
plot(sim_me_phyloXY$mcmc_results[[1]])
```

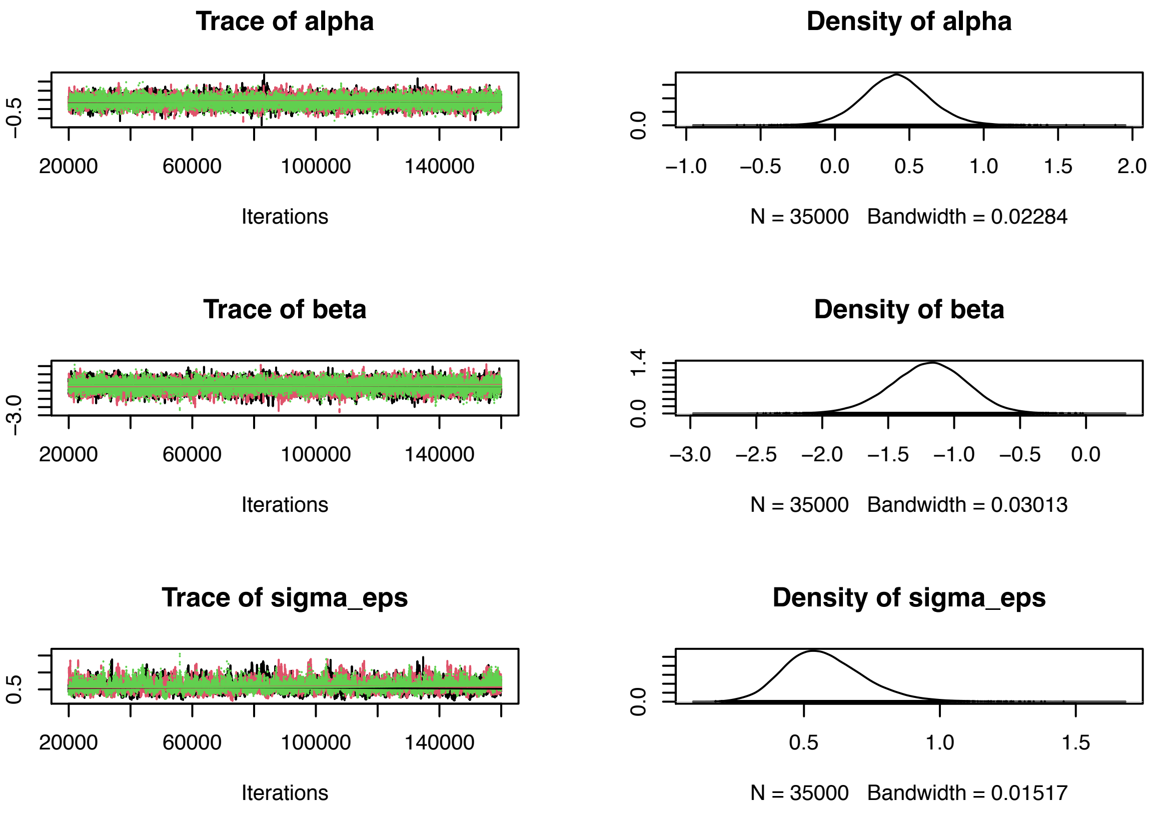

Fitting the model to real data

With simulation, we demonstrated the ability of our model to provide relatively unbiased estimates of parameters of interest when the true data-generating structure follows the assumptions of the model. Now, assuming the real data arose in a similar fashion, we can apply our model to real data. We allowed for the extra complexity of uncertainty in the phylogenetic tree in this analysis. The following is our model code written in JAGS (Plummer 2016).

```
social.maturity.model <- "model {
  # measurement error model for a logit-linear regression with known
  #correlation structure
  # Y is the number of species (Nspec) x 1 vector of TRUE logit
  #survival probs - D was observed.
  #X is Nspec x 1 vector of TRUE body length measurements - W was
  #observed.
  # TAUY/TAUX accounts for phylogenetic similarities in errors.
  Y[1:Nspec] ~ dmnorm(meanY[], TAUY[,])
  X[1:Nspec] ~ dmnorm(meanX[], TAUX[,])

  for(i in 1:Nspec){
# linear relationship between logit-survival and body length
# X[i] is true (unobserved) body length for species i
```

```
    meanY[i] <- alpha + blength*X[i]
    # account for measurement error (ME) in X
    # W[i] is the observed overall body length for species i
    # (here, average is used) - provided as data
    # comes from a normal distribution centered at with KNOWN
    # precision = to SE(Xbar)
    # ME precision assumed iid (i.e., correlation in measurements among
    #species due to phylogenetic similarities)

    W[i] ~ dnorm(X[i], tau_u[i])
    tau_u[i] <- pow(sigma_u[i], -2) # sigma_u[i] provided as data
    # here, we are assuming that the overall body lengths for each species
    # come from the same Normal population distribution (i.e., they are
    #exchangeable)

    meanX[i] ~ dnorm(mu_x, tau_x)

     # assume ME in X independent from ME in Y
    # D[i] is the observed body length for species i - provided as data
    # account for ME in Y - use Ferrari & Cribari-Neto (2004)
    # parameterization of the Beta distribution
    # transform true latent logit(survivalprob) to prob scale - p[i] latent
    # true survival probability
    p[i] <- ilogit(Y[i])

    # assume each observed probability ilogit(D[i]) is centered around p[i]
    # with variance set by phi[i], which is alpha[i] + beta[i] from
    #beta_loss function
    # D[i], phi[i] provided as data

    D[i] ~ dbeta(p[i]*phi[i], (1-p[i])*phi[i])
  }

# priors for precisions on populations
    tau_eps ~ dgamma(.001,.001)
    sigma_eps <- 1/sqrt(tau_eps)
    tau_x ~ dgamma(.001,.001)
    sigma_x <- 1/sqrt(tau_x)

# prior mean for distribution of average body lengths among species
    mu_x  ~ dunif(100, 700)

# priors for regression coefficients
    alpha ~ dnorm(0,0.5)
```

```
    blength ~ dnorm(0,0.5)

# Account for phylogenetic structure in Xs and Ys
    # Tree variance-covariance matrix construction for Pagel's lambda,
    # assuming equal prior probability for each tree.  NTree is the
    #number of candidate phylogenetic trees.
    for(l in 1:NTree){
      t[l] <- 1/NTree
    }
    L ~ dcat(t[])

    # prior for Pagel's lambda
    # A is the array of variance-covariance matrices constructed from the
    # candidate phylogenetic trees.  ID is the identity matrix.
    lambda ~ dunif(0,1)

    Mlam <- lambda*A[, , L] + (1-lambda)*ID
    TAUY <- tau_eps*inverse(Mlam)
    TAUX <- tau_x*inverse(Mlam)
}"
```

We used `runjags` (Denwood 2016) to fit the model.

```
#Code for using pinniped data

# specify the number of variance-covariance tree matrices and species
NTree <- 100
nspp <- 12
# initialize empty list
phylo <- array(NA, dim =c(nspp,nspp,NTree))
# specify path to data file with each tree in its own CSV file.
# this will list the file names.
path <- "~/Desktop/Complete models/vcv/"
tree_files <- list.files(path, pattern = "csv")

for(i in 1:100){
  # read each tree into list
  tree <- read.csv(paste0(path,tree_files[i]))
  tree <- tree[c(1,5:15), c(2, 6:16)]
  # scale tree
  tree <- tree/max(tree)
  tree <- as.matrix(tree)
  # store in ith position in list or [,,ith] position in 3-D array
  phylo[, , i] <- tree
}
```

```r
#Read in pinniped data for the age at social maturity
setwd("~/Desktop/Complete models")
df_socmat <- read.csv("pinniped_surv_socmat.csv")

surv_dat <- df_socmat[,c("Social.Mature.Survival", "SoM.Surv.SE")]

ab_surv <- apply(surv_dat, 1, function(x){
  beta_loss(mean = x[1], se = x[2])
})

# take from list to data frame
ab_surv <- do.call(rbind, ab_surv)
ab_surv

phi <- apply(ab_surv, 1, sum)

inits <- function(){ list(alpha = rnorm(1, 0, 5),
                          blength = rnorm(1, 0, 5),
                           lambda = runif(1, 0,1),
                          X = rep(300, 12),
                          Y = rep(0.5, 12)
)}

params_me_phylo <- c("alpha", "blength", "sigma_eps", "sigma_x", "p","mu_x",
                     "X", "lambda")

data_me_tree <- list(Nspec = 12, #number of species in dataset
                     NTree = 100,
                     #vector of standard length estimates
                     W = df_socmat$Standard.Length,
                     #vector of estimated SE
                     sigma_u = df_socmat$SE.of.Std.Length,
                     #vector of survival rate estimates on prob scale
                     D = df_socmat$Social.Mature.Survival,
                     #hyperparmeter values for prior on survival prob
                     phi = phi,
                     A = phylo,
                     ID = diag(12))

socmat_fit <- run.jags(model = social.maturity.model,
                      monitor = c(params_me_phylo),
                      data = data_me_tree,
                      n.chains = 3,
                      thin = 5,
```

```
                     burnin = 50000,
                     sample = 100000,
                     inits = inits)
```

References

Denwood, Matthew J. 2016. "runjags: An R Package Providing Interface Utilities, Model Templates, Parallel Computing Methods and Additional Distributions for MCMC Models in JAGS." *Journal of Statistical Software* 71 (9): 1–25. https://doi.org/10.18637/jss.v071.i09.

deVillemereuil, Pierre, Jessie A Wells, Robert D Edwards, and Simon P Blomberg. 2012. "Bayesian Models for Comparative Analysis Integrating Phylogenetic Uncertainty." *BMC Evolutionary Biology* 12 (102): 1–16. http://www.biomedcentral.com/1471-2148/12/102.

Gelman, Andrew, John B. Carlin, Hal S. Stern, David B. Dunson, Aki Vehtari, and Donald B. Rubin. 2014. *Bayesian Data Analysis*. Third Edition. Florida, USA: Taylor & Francis Group.

Plummer, Martyn. 2016. *Rjags: Bayesian Graphical Models Using Mcmc*. https://CRAN.R-project.org/package=rjags.

R Core Team. 2019. *R: A Language and Environment for Statistical Computing*. Vienna, Austria: R Foundation for Statistical Computing. https://www.R-project.org/.
